# Editorial: Post-Traumatic Osteoarthritis After Meniscus Injury

**DOI:** 10.3389/fbioe.2022.893800

**Published:** 2022-04-05

**Authors:** Jay M. Patel, Tammy L. Haut Donahue, Fabio Galbusera, Björn H. Drews, Andreas M. Seitz

**Affiliations:** ^1^ Department of Orthopaedics, Emory University, Atlanta, GA, United States; ^2^ Atlanta VA Medical Center, Decatur, GA, United States; ^3^ Department of Biomedical Engineering, University of Memphis, Memphis, TN, United States; ^4^ Schulthess Klinik, Zürich, Switzerland; ^5^ St. Vinzenz Hospital, Pfronten, Germany; ^6^ Institute of Orthopaedic Research and Biomechanics, Ulm University Medical Center, Ulm, Germany

**Keywords:** meniscus, osteoarthiritis, biomechanics, PTOA, meniscus repair

The menisci are fibrocartilaginous discs that are vital to load distribution and function of our knee joints. Due to their propensity for injury and relative lack of self-healing, these functions are often disrupted following traumatic injury. The result is the progression of post-traumatic osteoarthritis (PTOA), a huge burden on the population. In this Frontiers Research Topic, we bring together relevant contributions on various aspects of meniscal injury and PTOA progression, including meniscal function, metrics for studying PTOA, and repair considerations to prevent PTOA progression.

The meniscus has long been considered vital to distributing tibiofemoral loads in the joint (Lee et al., 2006). Sukopp et al. reviewed this literature, confirming that impaired knee joints (tears, meniscectomy, etc) consistently led to increased stresses. However, this review importantly noted that loading protocols were quite variable, and that future evaluation of load distribution may need better control to allow comparison across the field. Beyond load distribution, another meniscal function that has been up for debate is shock absorption. Classically, shock absorption had been cited as a key function of the menisci, until about 10 years ago (Andrews et al., 2011), when a nearly decade-long drop in Pubmed results for a search on “meniscus, shock absorption” is noted. Seitz et al. performed a robust evaluation of loss factors for intact, torn, and removed conditions, clearly demonstrating that there is indeed a shock absorbing function of the meniscus. Another important function of menisci is lubrication; de Roy et al. performed a study showing that both root injuries and meniscectomy do not affect whole joint friction. In the absence of changes to joint friction, patients may not “feel” a change that otherwise may result from catching or locking, and the initiation of PTOA under such conditions is likely associated with other functional deficits.

The effects of meniscal injury and removal on time-zero mechanics, as described previously, have been clearly linked to PTOA progression, especially in animal models involving destabilization of the medial meniscus (DMM) (Glasson et al., 2007; Bansal et al., 2020). Conversely, Haut Donahue et al. performed a morphological study on meniscus, cartilage, and bone health following a closed-joint traumatic injury model (resulting in ACL rupture), showing that even if the ACL was reconstructed after this injury, that the meniscus and cartilage still saw effects of damage. These new closed-joint models may better recreate the *in vivo* clinical situation, instead of the standard surgical DMM models. These models would also benefit from more standardized outcome metrics and guidance documents (Pfeifer et al., 2015), to truly evaluate the state of PTOA following meniscus injury, repair, and/or replacement. Trivedi et al. performed a systematic review of the outcome metrics of PTOA that were included in meniscus repair and replacement *in vivo* studies, showing that joint health is not always evaluated even if it is vital to determining the success of these approaches. Furthermore, new techniques are also being developed to analyze the state of articular cartilage, and could serve to identify early markers of PTOA that are not visible with current clinical imaging or arthroscopy. Gao et al. developed a novel confocal Raman microspectroscopy approach to spatially map glycosaminoglycan (GAG) loss, a technique that does not require tissue dissection or sampling, and that could be readily adapted toward non-destructive and potentially *in vivo* application (Gaifulina et al., 2021). Finally, in addition to establishing novel and consistent metrics for PTOA assessment, the identification of meniscal tears before PTOA initiation may be key to treating patients. Tack et al. utilized an MRI database from the Osteoarthritis Initiative, using a new neural network to identify meniscal tears. Combining these analytical advancements with technological innovations will give the field routes to identify both meniscal injuries and early markers of PTOA.

The multi-functional attributes of the meniscus, their influence on the progression of degenerative changes, and methods to evaluate early PTOA are all certainly of interest to the field. These findings, both inside and outside of this Research Topic, can also help to inform various repair and replacement techniques. For example, medial meniscus root tears are common yet present a huge mechanical burden due to a loss in load distribution and shock absorption (as noted by Sukopp et al., Seitz et al.). Repair of these root tears with transtibial techniques has shown promise (Pache et al., 2018); however, anatomic positioning of the repair site is also crucial. Floyd et al. show that non-anatomic repair of posterior medial meniscus root tears has consequences, including symptomatic extrusion, which may accelerate PTOA progression. Beyond root tears, research regarding the healing of the meniscal body post-injury is becoming increasingly common (Bansal et al., 2021), with the phrase “Save the Meniscus” commonly appearing in social medial and journal articles. Meniscus tear management often relies on the zone of tear; outer meniscus tears are repaired with suture and inner meniscus tears are typically treated with removal of the torn tissue (partial meniscectomy). This difference is often attributed to vascularity, however an opinion piece by Patel theorizes other factors that may be involved: circumferential disruption, obstruction by the dense matrix, and other joint factors (including inflammation). A couple of these factors were investigated by Andress et al., where anti-inflammatory modulation via compressive or tensile loading was confirmed with RNA-sequencing, verifying the role that loading (or lack thereof with disruption) may have in meniscal repair. Moreover, this contribution represents an exciting trend in meniscus research, combining omics and engineering to simultaneously study mechano-transduction and identify novel therapeutic targets. Finally, while identification of these therapeutic targets is important, implementing them *via* new scaffold technologies is also paramount. Dorthé et al. utilized a new technique called pneumatospinning, allowing the formation of thicker fiber mats that support fibrocartilage growth, and advancement over other nanofibrous scaffold fabrication techniques. This proof-of-concept study certainly warrants future exploration and may represent a method to better integrate with the native meniscal tissue.

As the meniscus research field continues to gain traction, this Frontiers Research Topic highlights new techniques in the field (Haut Donahue et al., Gao et al., Tack et al., Andress et al., Dorthé et al.), reviews the current state (Sukopp et al., Trivedi et al.), and establishes and/or challenges paradigms (Seitz et al., de Roy et al., Floyd et al., Patel), encompassing aspects of biomechanics, engineering, biology, and clinical management.
